# Genetic associations between feed efficiency traits and breeding goal traits in Nordic Holsteins

**DOI:** 10.3168/jdsc.2025-0898

**Published:** 2025-12-01

**Authors:** R.B. Stephansen, B.G. Poulsen, J. Lassen, J. Jensen

**Affiliations:** 1 Center for Quantitative Genetics and Genomics, Aarhus University, 8000 Aarhus C, Denmark; 2 Viking Genetics, Assentoft, 8960 Randers, Denmark

## Abstract

Genetic selection is a viable tool for improving feed efficiency due to its cumulative nature. However, it has not been investigated how selection for feed efficiency affects other breeding goal traits. This work investigated associations between the feed efficiency–related traits BW (representing energy requirement for body maintenance), genetic residual feed intake (gRFI; representing deviations from expected feed intake calculated from milk yield, maintenance, and changes in BW), genetic Feed Saved (gFS; representing deviations from expected feed intake calculated from milk yield, and changes in BW), and all breeding goal traits in the Nordic Total Merit (NTM) index. Phenotypic data were collected from 5,104 cows across 8 commercial herds using the Cattle Feed InTake system, and GEBVs for feed efficiency were estimated using the single-step genomic BLUP methodology. Genetic correlations were approximated using Pearson correlation between GEBVs for different traits. The genetic correlations were weakly negative between BW and NTM (−0.15 to −0.18) or longevity (−0.13 to −0.15), and it was not associated with the yield index. The genetic correlations were generally moderate between BW and conformation traits such as stature and chest width but not with dairy form. The genetic correlations for gRFI in different lactation stages were weakly to moderately correlated with the yield index (−0.23 to 0.21) and weakly correlated with functional traits (−0.10 to 0.10; e.g., mastitis, female fertility). Furthermore, gRFI in early lactation of multiparous cows was weakly and unfavorably correlated with longevity (−0.14). The genetic correlations between gFS and yield, functional traits, and longevity were low (−0.13 to 0.16). However, the moderate negative genetic correlation between gFS and frame (−0.37 to −0.28) indicated that more efficient cows have a smaller frame. In conclusion, our results indicated that feed efficiency traits such as gRFI and gFS can be integrated into breeding goals with limited adverse effects on yield, functional traits, and longevity. Selection for lower BW may enhance longevity without compromising yield.

## Highlights

• gRFI showed no clear associations with yield, functional traits, and longevity.
• gFS showed no clear associations with yield, functional traits, and longevity.
• gFS align well with Nordic Saved Feed index, included in the Nordic breeding goal.
• Selecting for defined feed efficiency traits did not show trade-offs in breeding goal traits.
• Lower BW is linked to improved longevity and has no effect on yield.

## Graphical Abstract

**Figure:**
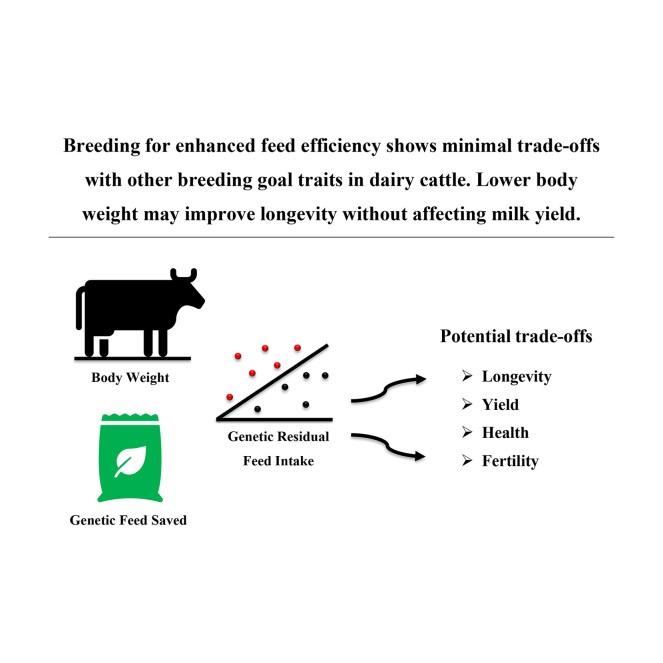
**Summary:** Enhancing feed efficiency in dairy cattle is essential for a more sustainable milk production. However, understanding how genetic selection for feed efficiency affects other important traits (e.g., production, health, and fertility) is not well explored. This study explored that for the feed efficiency traits: body weight (BW), genetic residual feed intake (gRFI), and genetic Feed Saved (gFS). Using data from >5,100 cows in commercial herds, we found that BW was slightly unfavorably associated with productive lifetime and showed no benefit for 305-day yield, suggesting that selecting smaller cows may enhance lifespan. Both gRFI and gFS showed low correlations with 305-day yield and functional traits, indicating minimal trade-offs. However, early lactation gRFI in multiparous cows had a slight negative correlation with longevity, warranting further investigation. The gFS index showed strong alignment with the current Nordic Saved Feed index and selection for gFS is unlikely to negatively affect other traits in the breeding goal.

The global dairy production is expected to increase by 1.8% each year between 2025 and 2034 (OECD/FAO, 2025). The production of these additional dairy products will likely result in additional feed consumed by dairy cows. This poses both financial and sustainability challenges, because feed is the major contributor to both the variable costs and the carbon footprint of dairy production (Capper et al., 2009). Therefore, it would be beneficial if more milk was produced with less feed (i.e., if the dairy cows were more feed efficient; VandeHaar et al., 2016). Recently, multiple genetic evaluation centers have introduced breeding values of feed efficiency and, in some countries, incorporated them into national breeding goals (de Jong et al., 2019; Jamrozik et al., 2021; Stephansen et al., 2021; Van Raden et al., 2021). However, most genetic evaluation centers have a limited amount of data on individual feed intake that often is collected in a limited period of an animal's life. This may favor selection of short-term efficiency, which is at risk of being unfavorably associated with female fertility and productive lifetime (Friggens et al., 2017). On the contrary, selection for long-term efficiency may favor genes associated with nonproductive life functions such as immune response and adequacy of body reserves (Martin et al., 2021). Ideally, recording schemes of genetic evaluations for feed efficiency should cover the whole productive lifetime, but such studies are generally not conducted on a large scale because of high recording costs for individual feed intake.

Most genetic evaluation centers have introduced breeding values for feed efficiency through the Feed Saved index (Feed Saved; Jamrozik et al., 2021; Stephansen et al., 2021; Van Raden et al., 2021; Abdalla et al., 2024). Pryce et al. (2015) defined Feed Saved as the sum of feed saved from BW dependent maintenance requirements and an improved metabolic efficiency through residual feed intake (**RFI**). However, knowledge on how Feed Saved and its component traits (e.g., DMI, ECM, BW) are correlated with other breeding goals traits is limited, such as female fertility and udder health. Such knowledge is important for decision making by breeders and for genetic evaluation centers when deciding the emphasis on Feed Saved in a breeding goal. Therefore, we aimed to explore correlations between breeding values for Feed Saved and its component traits, BW, and RFI, and the index traits in the Nordic breeding goal Nordic Total Merit (**NTM**; https://nordicebv.info/), such as yield traits (e.g., milk production) and functional traits (e.g., female fertility, mastitis) both across and within lactation.

The phenotype data were obtained from 8 commercial Danish Holstein (**HOL**) herds that had the Cattle Feed InTake (**CFIT**) system installed (Borchersen, 2014; Borchersen et al., 2017; Lassen and Borchersen, 2020). The CFIT system is based on 3-dimensional imaging and the use of artificial intelligence to predict the amount of feed eaten by an individual cow and the cows' identification, which is different from scale-based methods, but has been shown to provide similar breeding decisions for feed efficiency (Stephansen et al., 2025a). Lassen et al. (2023) and Stephansen et al. (2025a) provide detailed descriptions of the farms and the data preprocessing used to generate cow-level weekly averages for DMI and BW. The same data editing criterion used in Stephansen et al. (2025a) was applied, which resulted in a total of 5,104 unique HOL cows with 242,671 weekly feed intake records in 9,400 lactations. In addition, 49,290 monthly test-day records from 12,388 lactations were included in the analysis. There were more lactations with phenotypes for milk yield than for the CFIT phenotypes because we included milk records that were obtained before CFIT was installed. The data from CFIT were collected between August 13, 2020, and September 13, 2024. To standardize the energy output in milk, we calculated ECM in accordance with the equation of Sjaunja et al. (1990). Based on Stephansen et al. (2025a), we regarded genetic residual feed intake (**gRFI**) as genetically different in early and mid to late lactation. For simplicity, we therefore defined a truncation point at 10 wk in milk (70 DIM), grouping breeding values summed over early lactation (early), the remaining part of lactation (rest), or the total lactation (all).

The initial pedigree originated from the Danish cattle database, and it was provided by SEGES Innovation (Skejby, Denmark). The pedigree was pruned for 10 generations using the DMU trace software (Madsen, 2012) by tracing ancestors of cows with data (5,104) and the most recent complete birth year of candidate bulls, which was born in 2023 (3,084). The resulting pedigree contained 20,744 HOL animals. We assigned genetic groups to animals that had missing parentage information using the same procedure as Stephansen et al. (2025a). The genotype data were provided by Nordic Cattle Genetic Evaluation (Skejby, Denmark). Most animals were genotyped with the 50k Illumina Bovine SNP50 and missing genotypes had been imputed before receival, following the standard Nordic Cattle Genetic Evaluation procedure (NAV, Skejby, Denmark). The genotype data received contained 46,342 SNPs. After removal of loci with minor allele frequencies smaller than 0.01, the genotype data contained 43,673 SNPs. In total, 13,218 animals in the pruned pedigree had genotype information. To calculate the combined relationship matrix of genotyped and nongenotyped animals, we used (Aguilar et al., 2010; Christensen and Lund, 2010) the following equation:

$$H^{-1}=A^{-1}+\left(\begin{array}{cc} 0 & 0\\ 0 & {\left(\omega G+\left(1-\omega \right)A_{22}\right)}^{-1}\;-\;A_{22}^{-1} \end{array}\right),$$

where **H**^−1^ is the inverse of the combined pedigree and genomic relationship matrix, **A**^−1^ is the inverse of the pedigree relationship matrix, **G** is the genomic relationship matrix, *ω* is the relative weight of the polygenetic effect (*ω* = 0.8), **A**_22_ is the part of the pedigree relationship matrix with genotyped animals, and $\;A_{22}^{-1}$ is the inverse of **A**_22_. We constructed the **G** matrix using method 1 from VanRaden (2008) in the *invgmatrix* software by Su and Madsen (2011).

We used the estimated (co)variance components for DMI, ECM, and BW of primiparous or multiparous cows from the 6-variate model in Stephansen et al. (2025a). From these covariance components, we derived the covariance matrices for gRFI and ΔBW using the lactation sum approach described in Stephansen et al. (2025a). A brief description of the 6-variate model using the pedigree-based relationship matrix is as follows:

**y** = **Xb** + **Mh** + **Za** + **Wpe** + **e**,

where **y** is the vector of phenotypes with subvectors of repeated measurements of DMI, ECM, and BW in 2 parity groups (primiparous and multiparous); **b** is the vector of fixed regressions with a fourth-order Legendre polynomial regression on weeks in milk and nested within herd × parity group (first, second, third, and fourth +), and in primiparous lactations a second-order Legendre polynomial regression on age at calving, and fixed effect of herd × CFIT algorithm; **h** is the vector of random effects for herd × year × test-week; **a** and **pe** are the vectors of random regressions for random additive genetic and permanent environmental effects of cows, using second-order Legendre polynomials (intercept, linear, and quadratic), with subvectors for each trait × parity group, respectively; **e** is the vector of random residual effects with subvectors of all traits in the 2 parity groups included in the analysis, assuming a homogeneous residual variance, and **X**, **M**, **Z**, and **W** are design matrices. To estimate GEBVs for all traits we applied the variance components of the multivariate model from the Gibbs sampler together with **H**^−1^ in a single-step genomic BLUP model using the DMU5 module with the preconditioned conjugate gradient computation method (Madsen and Jensen, 2013; Madsen, 2018).

The additive genetic correlations were based on the (co)variance parameters estimated in Stephansen et al. (2025a) for BW, gRFI, and genetic Feed Saved (**gFS**; Table 1). Results show moderately high genetic correlations between parity groups of gRFI in mid to late lactation (0.46 ± 0.10), whereas this correlation is moderate in early lactation (0.24 ± 0.14). Body weight yielded high additive genetic correlations between parity groups (>0.85 ± 0.02 to 0.03), which is consistent with findings by both Lidauer et al. (2019) and Stephansen et al. (2023). Genetic Feed Saved, calculated as proposed by Khanal et al. (2022), showed moderately genetic correlations across parity groups for all lactational traits (0.50 to 0.65 ± 0.06 to 0.09). As expected, the additive genetic correlations between gRFI and BW was zero by the definition of gRFI. Within parity group additive genetic correlations indicated that gRFI in early lactation was moderately to highly correlated with later lactation segments (0.37 to 0.72 ± 0.06 to 0.14). Similarly, gFS showed moderately to high genetic correlations (0.64 to 0.82 ± 0.04 to 0.08) from early to later lactation segments. Khanal et al. (2022) also observed this pattern, although their study only investigated the period from 50 to 200 DIM. In general, Table 1 shows that traits defined from 11 to 45 wk in milk have higher correlations with traits defined from all weeks in milk compared with traits defined in early lactation, following the expectation as the early lactation period only corresponds to 22% of the whole lactation.

**Table 1:** The posterior means additive genetic correlations between gRFI, BW, and gFS in early lactation (1–10 wk in milk), the rest of the lactation (11–45 wk in milk), and all weeks in milk^1^

Item	Multiparous								
	gRFI_early_	BW_early_	gFS_early_	gRFI_rest_	BW_rest_	gFS_rest_	gRFI_all_	BW_all_	gFS_all_
Primiparous									
gRFI_early_	**0.24**	0.00	0.77	0.55	−0.06	0.34	0.72	−0.04	0.45
BW_early_	0.00	**0.85**	0.62	−0.03	0.96	0.69	−0.01	0.98	0.72
gFS_early_	0.75	0.55	**0.50**	0.42	0.57	0.72	0.56	0.58	0.82
gRFI_rest_	0.37	0.00	0.29	**0.46**	0.00	0.64	0.96	−0.01	0.62
BW_rest_	0.01	0.90	0.62	0.00	**0.91**	0.74	0.00	1.00	0.75
gFS_rest_	0.27	0.62	0.64	0.63	0.73	**0.64**	0.62	0.74	0.99
gRFI_all_	0.63	0.01	0.41	0.94	0.00	0.59	**0.46**	0.00	0.64
BW_all_	0.00	0.94	0.65	0.00	1.00	0.75	0.00	**0.91**	0.75
gFS_all_	0.57	0.61	0.78	0.61	0.72	0.98	0.59	0.72	**0.65**

1 On the diagonal are additive genetic correlations between primi- and multiparous parity groups (posterior SD: 0.02 to 0.14), below the diagonal are additive genetic correlations for primiparous lactation (posterior SD: 0.00 to 0.14), and above the diagonal are additive genetic correlations for multiparous parity groups (posterior SD: 0.00 to 0.09). gRFI = genetic residual feed intake; gFS = genetic Feed Saved.

These findings highlight the importance of considering the stage of lactation when evaluating breeding values for feed efficiency and constructing composite traits. However, the lower genetic correlations in early lactation indicate that additional attention is needed to ensure that improvements in feed efficiency are consistent throughout the entire lactation period to enhance the overall lifetime efficiency. Ideally, genetic correlations were estimated between the efficiency traits (gRFI, gFS, and BW) and traits like fertility and disease resistance. However, the current dataset does not enable accurate estimation of such genetic correlations, and we aimed to make preliminary investigations using correlations between predicted breeding values. Here, Laloë et al. (1996) showed that differences between genetic and index correlations may arise due to connectedness among individuals, precision of estimation, and the structure of the data, meaning that index correlations do not always reflect genetic correlations. Therefore, our findings should be supported by future studies when more data are available. We calculated correlations among GEBVs for the defined efficiency metrics in this study with GEBVs from NAV routine genetic evaluation, using cows with data, and Nordic candidate HOL bulls born in 2023. For the feed efficiency traits, gRFI and gFS, we reversed the scales of GEBVs, meaning higher level of breeding values means more efficient animals.

The feed efficiency trait BW, representing weight dependent maintenance requirements, had small and negative correlations with the NTM across both parity and animal groups (ranging from −0.15 to −0.18; Figure 1). This suggests that selection for NTM is expected to indirectly favor lower BW. Genetic correlations between BW and the yield index were generally low, where the Nordic Yield index has negative emphasis on fluid yield and positive emphasis on milk solids (NAV, 2024), and most correlations to functional traits were near zero (−0.13 to 0.00). In addition, the numerically highest and favorable correlations were between BW and young stock survival (−0.13 to −0.11). These results indicate no yield benefits from increased BW and no obvious trade-offs with functional traits.

**Figure 1:**
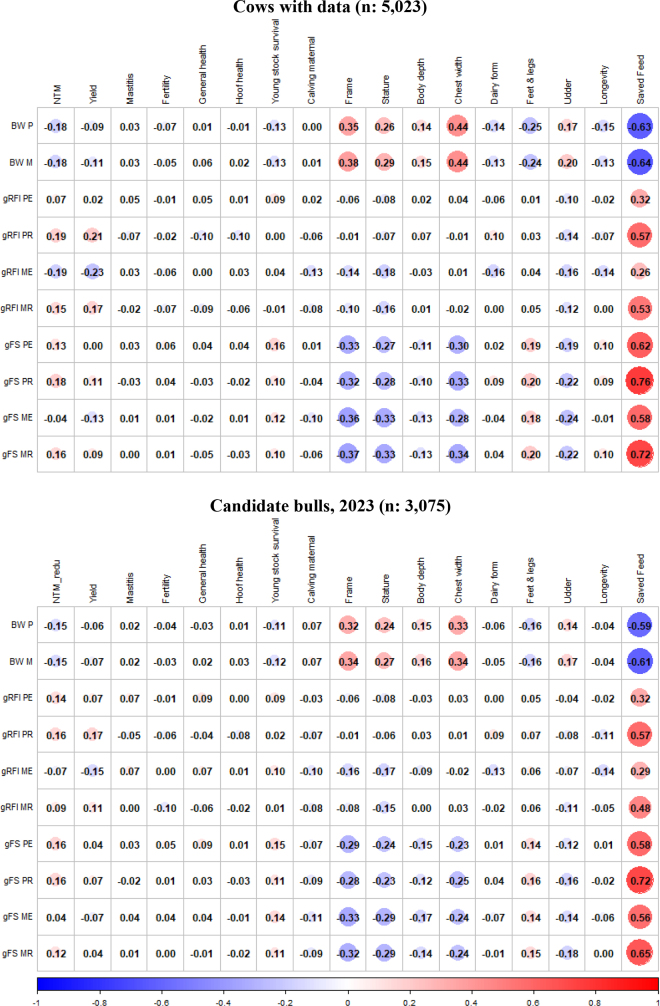
Index correlations between GEBV for feed efficiency traits, estimated using genetic parameters from Table 1, and GEBV for breeding goal traits from NAV routine genetic evaluation. For gRFI and gFS, the scale has been flipped, meaning higher breeding values are more efficient dairy cows. gRFI = genetic residual feed intake; gFS = genetic Feed Saved; PE = primiparous early lactation period; PR = primiparous rest of lactation period; ME = multiparous early lactation period; MR = multiparous rest of lactation period. Significant correlations (*P* < 0.001) had an absolute numeric correlation value of a minimum of 0.05 and a median of 0.14 across cows with data and candidate bulls. BW P = BW primiparous; BW M = BW multiparous.

As expected from Lidauer et al. (2019), BW showed mostly moderate and positive correlations with the conformation trait frame and its components—stature, body depth, and chest width (0.15 to 0.44)—but not with dairy form (Figure 1). This partially aligns with the US body weight composite index, although the US model includes a positive association with dairy form (Holstein Association USA, 2016), which was not observed here. Additionally, BW was negatively correlated with longevity for cows with data (−0.13 to −0.15) and HOL candidate bulls (−0.04), consistent with the routine Nordic evaluation of Saved Feed for HOL, where BW is negatively associated with longevity (−0.20; Stephansen et al., 2025b).

For gRFI, genetic correlations with yield ranged from absent to moderate (−0.23 to 0.21), indicating that selection for improved metabolic efficiency is expected to have limited effect on yield (Figure 1). A positive correlation between gRFI and NTM (0.09 to 0.19) was observed from 10 to 45 wk in milk, reflecting the indirect inclusion of gRFI in NTM via the Saved Feed index (index correlation of 0.30; Stephansen et al., 2025b). Correlations between gRFI and functional traits ranged from absent to low (−0.10 to 0.10), suggesting selection for gRFI is expected to have minimal trade-offs in functional traits. Similarly, correlations with conformation traits were low (−0.18 to 0.09), indicating that gRFI is largely independent of body conformation information (Figure 1). Notably, early lactation gRFI in the multiparous lactation group showed a low unfavorable correlation with longevity (−0.14), warranting further investigation into its biological basis and implications for selection, as this is an unfavorable correlation.

The current Nordic Saved Feed index, which is incorporated into NTM and has a reported index correlation of 0.30 to the breeding goal (Stephansen et al., 2025b), showed moderate to high correlations with gFS GEBVs (0.56 to 0.76), particularly from 10 wk in milk onward (≥0.65). However, gFS differed from the Saved Feed index by using regressions not estimated from the variance component estimates and by combining lactational breeding values of Saved Feed with 1/3 emphasis on primi- and 2/3 emphasis on multiparous lactations. Genetic correlations between gFS and yield (−0.13 to 0.11), functional traits (−0.11 to 0.16), and longevity (−0.06 to 0.10) were low (Figure 1), consistent with previous findings (Abdalla et al., 2024; Stephansen et al., 2025b). These results suggest that selection for gFS is unlikely to negatively affect important breeding goal traits in the HOL population of the Nordic countries. However, a moderate negative correlation with frame (−0.37 to −0.28) indicates that more gFS feed-efficient cows tend to have smaller bodies.

In conclusion, this study highlights differences in cow ranking for feed efficiency across lactation stages when using gRFI or gFS. Both feed efficiency metrics showed low correlations with yield and functional traits, with early lactation gRFI in multiparous cows showing a weak negative correlation with longevity, that is unfavorable. Body weight was not associated with yield but was negatively correlated with longevity, supporting that selecting for lower maintenance requirements may enhance cow lifespan and feed efficiency jointly. These findings underscore the importance of refining feed efficiency metrics and understanding their genetic architecture to optimize breeding strategies in modern dairy populations.
